# Fellowships, Grants, & Awards

**Published:** 2005-10

## Outstanding New Environmental Scientist (ONES) Award

An essential element of the mission of the NIEHS is the support and career promotion of the future generation of exceptionally talented and creative new scientists who will further the understanding of the impact of environmental exposures on human health. The NIEHS supports a number of training and fellowship programs for pre- and postdoctoral training, and mentored career development awards for faculty in the early stages of their career development. Primary among these are the Ruth Kirschstein National Research Service Awards for pre- and postdoctoral training, the Career Development Awards for clinically trained scientists (K08 and K23), and the Mentored Quantitative Research Career Development Awards to support the career development of scientists with quantitative and engineering backgrounds who wish to integrate their expertise with biomedicine. In addition, in 1999 the NIEHS instituted the Transition to Independent Positions Program to address the progression of individuals from postdoctoral positions to faculty positions. In this career development award the individual applies for the grant while still in a postdoctoral position, and the grant for start-up funding is awarded at the institution where the candidate accepts the faculty position. However, even with these career development mechanisms in place, to fulfill its mission of assuring a cadre of productive environmental health science investigators for the future, NIEHS needs to initiate further imaginative programs to identify the best new biomedical investigators and facilitate their establishing vibrant, independent research programs in the environmental health sciences.

To identify outstanding scientists at the formative stages of their career and assist them in launching an innovative research program with a defined impact in the environmental health sciences, the NIEHS is establishing a program of R01 research grants intended for researchers who have not received their first R01 research grant. It is designed to be highly competitive, and only a limited number will be awarded per year.

Research programs supported by this announcement seek to promote career advancement of the most highly creative and promising new scientists who intend to make a long-term career commitment to research in the mainstream of the environmental health sciences, and bring innovative, ground-breaking research initiatives and thinking to bear on the problems of how environmental exposures affect human biology, human pathophysiology, and human disease.

The R01 applications in this program are distinguished from other R01 research grants in that the applications 1) incorporate a statement of career goals in the environmental health sciences; 2) include a discussion of previous research experience and achievements in addition to the research proposal; 3) may include active participation of an external advisory committee; 4) require demonstration of the commitment by the institution to actively support the research program development of the principal investigator (PI); and 5) include a separate budget specifically devoted to equipment and career enhancement activities.

Research projects proposed in response to this Request for Applications will be expected to have a defined impact on the environmental health sciences and be responsive to the mission of the NIEHS, which is distinguished from that of other Institutes by its focus on research programs seeking to link the effects of environmental exposures to the cause, mechanisms, moderation, or prevention of a human disease or disorder or relevant pathophysiologic process. For purposes of this announcement, all applications must focus on a specific human disease, dysfunction, pathophysiologic condition, or relevant human biologic process and propose to study a specific environmentally relevant toxicant. Examples of environmentally relevant toxicants include industrial chemicals or manufacturing byproducts, metals, pesticides, herbicides, air pollutants, and other inhaled toxicants, particulates or fibers, and fungal, bacterial, or biologically derived toxins. Agents considered nonresponsive include, but are not limited to, alcohol, chemotherapeutic agents, radiation that is not a result of an ambient environmental exposure, drugs of abuse, pharmaceuticals, and infectious or parasitic agents, except when these are disease co-factors to an environmental toxicant exposure to produce the biological effect.

Applicants involving animal exposures must include a justification of how the exposure paradigm is relevant to human exposure and clearly discuss the link between the exposure and the relevant human disease in the Background and Significance section of the application. The applicant should also discuss the potential for translating the research—applying the ideas, insights, and discoveries generated through the basic inquiry to the treatment or prevention of human disease. Applicants proposing epidemiologic research should address how the significant associations revealed in the studies could be confirmed in the laboratory setting.

The ONES program would be evaluated on a continuing basis by the NIEHS, to assess the impact of the program on the portfolio of the NIEHS as well as on the progression of the awardees' careers. Metrics to be used include, but are not limited to, publications (numbers and impact factors of publications); academic promotion of PIs; awards, invited talks at national/international symposia, students and postdoctorals trained in the PI's laboratory, and honors received by PIs; committee service by PIs; and subsequent grant support awarded. The design of the program evaluation will be determined by the Program Analysis Branch of the Division of Extramural Research and Training. PIs of awarded ONES grants must provide information for the evaluation and any subsequent program evaluations for up to 10 years after the award.

This funding opportunity will use the R01 Grant mechanism. This funding opportunity uses the just-intime budget concepts. It also uses the nonmodular budget format described in the PHS 398 application instructions (see http://grants.nih.gov/grants/funding/phs398/phs398.html). A detailed categorical budget for the "Initial Budget Period" and the "Entire Proposed Period of Support" is to be submitted with the application. For further assistance contact GrantsInfo, 301-435-0714 (telecommunications for the hearing impaired: TTY 301-451-0088) or by e-mail: GrantsInfo@nih.gov

Because the nature and scope of the proposed research will vary from application to application, the size and duration of each award will also vary. Although the financial plans of the NIEHS provide support for this program, awards pursuant to this funding opportunity are contingent on the availability of funds and the receipt of a sufficient number of meritorious applications.

Applications must be prepared using the most current PHS 398 research grant application instructions and forms. Applications must have a Dun & Bradstreet (D&B) Data Universal Numbering System number as the universal identifier when applying for federal grants or cooperative agreements. The D&B number can be obtained by calling 866-705-5711 or through the web site at http://www.dnb.com/us/. The D&B number should be entered on line 11 of the face page of the PHS 398 form.

The deadline for receipt of letters of intent is 20 November 2005, with 22 December 2005 the deadline for receipt of applications. The complete version of the RFA is available at http://grants.nih.gov/grants/guide/rfa-files/RFA-ES-05-005.html

Contact: Carol Shreffler, Division of Extramural Research and Training, National Institute of Environmental Health Sciences, P.O. Box 12233, EC-23, 111 T.W. Alexander Drive, Research Triangle Park, NC 27709 USA, 919-541-1445, fax: 919-541-5064, e-mail: shreffl1@niehs.nih.gov. Reference: RFA-ES-05-005

## Centers for Children’s Environmental Health and Disease Prevention Research

This initiative continues NIEHS’s and EPA’s intent to foster advances in children’s health by supporting innovative, state-of-the-art Research Centers examining the adverse health effects of environmental exposures among children. Both agencies are interested in reducing environmental risks to children and alleviating the societal burden of environmentally-induced disease/dysfunction.

Collaborative, multidisciplinary research approaches are required to explore the dynamic interaction of children and the environment. Centers are expected to have fully coordinated programs that incorporate exposure and health effects research to support the development and validations of novel health promotion strategies. A Center should identify a central theme or focus of its research effort so that the proposed projects are responsive to the specific research area of children’s environmental health included in this Center program.

The program emphasizes integration of basic and population sciences while utilizing a community-based participatory research (CBPR) approach. By bridging gaps between basic and clinical research and between institutional researchers and community members, this program aims to improve our knowledge regarding detection, treatment, and prevention of environmentally-induced disease/dysfunction in children.

The long-range goals of this program are 1) to stimulate new and expand existing research on the role of environment in the etiology of disease/dysfunction among children, 2) to develop novel effective intervention and prevention strategies, and 3) to promote translation of basic research findings into applied intervention and prevention methods, thereby enhancing awareness among children, their families, and health care practitioners regarding detection, treatment, and prevention of environmentally related diseases and health conditions.

A spectrum of scientific approaches is expected to create a truly multidisciplinary working environment where basic research can inform clinical research. These may include: 1) mechanistic research including pathophysiology of target-organ system; 2) toxicologic research; 3) molecular and cellular sciences; 4) clinical research; 5) public health research including epidemiology; 6) exposure assessment and remediation; 7) behavioral and social sciences; 8) cost/benefit; and 9) social policy research.

Each Center must produce a synergistic research environment that allows each research effort to share the creative strengths of the others. Each Center, by supporting interrelated projects and collaborating investigators, is expected to yield results beyond those achievable were each project pursued separately and without formal interaction among the participating investigators. The demonstration of synergy among the projects and the multidisciplinary nature of the work are critical components of this program. Ultimately, the expected outcomes of this research program include 1) the generation of cutting-edge science, 2) the development of local and national networks of children’s environmental health research professionals, and 3) peer-reviewed data relevant to better understanding vulnerability and risk. Expected outputs from each Center include 1) contributions to the scientific literature and 2) translational outreach and communication tools developed with and applicable for the effected community of concern.

For this RFA, NIEHS and EPA will accept applications focused on environmentally mediated disorders/dysfunctions of the nervous and/or endocrine systems. Applicants must study an environmental agent/chemical/stressor to which there is human exposure and the potential for *in utero* exposure. This includes any endocrine active chemical(s) or organic solvents, particulate matter (PM), pesticides, nutritional supplements, phytochemicals or metals; nutrition and social and cultural factors cannot be considered alone. Applicants are encouraged, however, to incorporate these factors in assessing the effects of previously described environmental exposures. These areas are of interest: 1) mental retardation, cerebral palsy, autism spectrum disorders, visual and or hearing impairment, attention deficit and hyperactivity disorders, and affective disorders; 2) delays or deficits in domains of neurodevelopment such as cognition, motor, sensory; 3) thyroid/pituitary dysfunction, puberty and sexual maturation, reproductive development, sexual dimorphic phenotypes, growth disorders or impairments including but not limited to childhood obesity; 4) reproductive outcomes such as preterm delivery, birth defects; 5) other outcomes associated with nervous and/or endocrine system disruption.

Each Center will propose an overall research mission and plan that is responsive to the objectives of the NIH Center Program. The application must contain a minimum of three research projects, including the first two listed below: 1) laboratory basic research project; 2) clinical research project; 3) other research project(s). At least one of the three projects must use the CBPR process as defined below. Applications lacking the first two projects and a project using the CBPR process will be considered nonresponsive and returned without review.

Each Center must also include the Community Outreach and Translation Core (COTC) and the Administrative Core. Applications lacking these two cores will be considered nonresponsive and returned without review.

Laboratory-based research projects may include mechanistic studies of environmental agents that contribute to adverse health outcomes in children as well as research that will improve our basic understanding of pathophysiology, molecular genetics, or cell biology of developmental processes. Basic mechanistic research may pertain to the disciplines of toxicology, cell and molecular biology, physiology, psychology, genetics, or other relevant fields, and methods may include animal models, *in vitro* systems, and/or human clinical specimens.

Clinical research is conducted with human subjects (or on material of human origin such as tissues, specimens, and cognitive phenomena) for which an investigator (or colleague) directly interacts with human subjects. Excluded are *in vitro* studies that utilize human tissues that cannot be linked to a living individual. For this RFA, two types of clinical research would be accepted: 1) patient-oriented research (mechanisms of human disease, therapeutic interventions, clinical trials); 2) epidemiologic and behavioral studies. Clinical research that explores gene and environmental interactions in risk of disease/dysfunction is highly encouraged

At least one other “research project(s)” must be proposed. The additional project(s) must be thematically related and integrated with the above two projects. For example: 1) Studies characterizing pathways of exposure; the magnitude, frequency, duration, and time-pattern activities that lead to contact in children and quantifying contact rates of children with exposure media, contaminant transfer efficiencies, and uptake rates in children; 2) research on behavioral factors that affect exposure and or the ability to reduce exposure; 3) research that characterizes the economic and social impact of children’s illnesses on society; 4) evaluation of how scientific information about children’s environmental health affects policy, social change, and changes in clinical and public health management of these diseases.

In CBPR, scientific inquiry is such that community members, persons affected by the health condition, disability, or issue under study, or other key stakeholders in the community’s health can be full participants in each phase of the work (conception–design–conduct–analysis–interpretation–conclusions–communication of results). CBPR is characterized by substantial community input in the development of the project.

Community refers to populations that may be defined by geography; race; ethnicity; sex/gender; sexual orientation; disability, illness, or other health condition; or to groups that have a common interest or cause, such as health or service agencies and organizations, health care or public health practitioners or providers, policy makers, or lay public groups with public health concerns. Community-based organizations may be involved in the research process as members or representatives of the community. Organizations as varied as tribal governments and colleges, state or local governments, independent living centers, other educational institutions such as junior colleges, advocacy organizations, health delivery organizations (e.g., hospitals), health professional associations, nongovernmental organizations, and federally qualified health centers are possible community partners. Each Center must include 1) Description of Cores and 2) a COTC and an Administrative Core.

A COTC is required to develop, implement, and evaluate strategies to translate and apply the scientific findings of the Center into information for the public, policy makers, and clinical professionals to use to protect the health of children. This must include personnel from one or more of the following areas: health educators, nurses, members of community or faith-based organizations, members of organizations that advocate for research and services pertaining to children’s health, members of professional societies of health care professionals, and state and local health departments or medical service organizations. Examples of activities considered responsive are creating training materials for health professions, developing new ways to disseminate research findings to the broad audience of stakeholders, and assessing community understanding of research results and plans for action. A Center will devote at least 10% of its budget to the COTC.

Each Center must include an Administrative Core unit to provide oversight, coordination, and integration of Center activities and establish an External Advisory Committee (EAC) to the Center Director. The EAC should consist of three to five scientists with expertise appropriate for the Center’s research focus, plus one representative from a community-based organization involved in community-based research. Representation from a state or local health department is also encouraged. At least 67% of Committee members should be from outside the grantee institution. The membership of the EAC must be approved by the funding agency. The EAC is to help evaluate the merit, value, and contribution of research projects and the relevance and importance of individual organizational elements to the overall goals of the Center. The membership of the EAC must be approved by the Participating Agencies postreview; names should not be submitted in the application. Individuals in senior leadership positions should provide intellectual, administrative, and scientific leadership for the Center and are critical to its overall effectiveness and evolution. These individuals should be in place and committed to a defined percent effort. Please submit only a description of proposed protocols and planned committee by representation and area of expertise. If awarded, you must provide an identifiable list of membership to the EAC for approval by the funding agencies.

Each Center may support other cores that provide a technique, service, or instrumentation that will enhance ongoing research efforts, such as animal resources, cell/tissue culture, pathology, biostatistics, molecular biology, neuropsychology, neuroimaging, analytical chemistry, exposure assessment, genotyping, and resequencing. Budgeted Center projects and external research projects may have access to these cores. The application should provide a total operational budget for each facility core together with the percentage of support requested from the Center grant. The application should explain the organization and proposed mode of operation of each core, including a plan for usage, priority setting, allocation of resources, and any applicable charge-back system.

Within the Center Program, PIs are encouraged to support training of new investigators within the proposed projects. New investigators should have a doctoral degree with < 8 years of postdoctoral experience at the time of application, and have demonstrated outstanding abilities in basic, clinical, or population-based research, such as postdoctoral research in an academic, industry, or government environment. However, years of clinical training will not count against the limitation. Ineligible individuals include current and former PIs on EPA STAR Grants or NIH research projects (R01), subprojects of program projects (P01), or Center Grants with research components (P50) or equivalent research grant awards. Applicants will be expected to devote at least 50% time and effort to the award and have a long-term commitment to research in the environmental health sciences.

This funding opportunity will use the NIH P01 award mechanism and the EPA’s Office of Research and Development, STAR Grant awards. As an applicant, you will be solely responsible for planning, directing, and executing the proposed project.

Applications must be prepared using the most current PHS 398 research grant application instructions and forms. Applications must have a D&B Data Universal Numbering System (DUNS) number as the universal identifier when applying for Federal grants or cooperative agreements. The D&B number can be obtained by calling (866) 705-5711 or through the web site at http://www.dnb.com/us/. The D&B number should be entered on line 11 of the face page of the PHS 398 form.

This funding opportunity uses the just-in-time budget concepts. It also uses the non-modular budget format described in the PHS 398 application instructions ªsee http://grants.nih.gov/grants/funding/phs398/phs398.html). A detailed categorical budget for the “Initial Budget Period” and the “Entire Proposed Period of Support” is to be submitted with the application. For further assistance contact GrantsInfo, 301-435-0714 (telecommunications for the hearing impaired: TTY 301-451-0088) for by e-mail: GrantsInfo@nih.gov.

The deadline for receipt of letters of intent is October 23, 2005, with November 24, 2005 the deadline for receipt of applications The complete version of the RFA is available at http://grants.nih.gov/grants/guide/rfa-files/RFA-ES-05-004.html.

Contact: Kimberly A. Gray, Division of Extramural Training and Science National Institute of Environmental Health Sciences, PO Box 12233, EC-21, 111 T.W. Alexander Drive, Research Triangle Park, NC, 27709 USA, 919-541-0293, fax: 919-316-4606, e-mail: kg89o@nih.gov; Chris Saint, National Center for Environmental Research, U.S. Environmental Protection Agency (8723R), 1200 Pennsylvania Ave. NW, Washington, DC 20460 USA, 202-564-6909, fax: 202-565-2448, e-mail: saint.chris@epa.gov; Nigel Fields, National Center for Environmental Research, U.S. Environmental Protection Agency (8723F), 1200 Pennsylvania, Ave. NW, Washington, DC 20460 USA, 202-343-9767, fax: 202-233-0677, e-mail: fields.Nigel@epa.gov. Reference: RFA-ES-05-004

